# The Relation of Amebiosis to Pyorrhea Alveolaris

**Published:** 1916-07

**Authors:** Julio Endelman


### THE RELATION OF AMEBIOSIS TO PYORRHEA ALVEOLARIS.

JULIO ENDELMAN, D.D.S.

It is less than two years since the dental profession was informed of the supposed pathogenic role played by a certain mouth protozoon in inflammations of the investing tissues of a tooth. In many quarters the claims of the investigators responsible for the diffusion of this theory were not only accepted in full, but in addition were so amplified as to overshadow for the time being the role of the many other pathogenic inhabitants of the mouth. To those who, up to that time, had made careful observations of the pathologic changes which characterize pyorrhea alveolaris, the idea of attributing the inflammatory reactions, in all instances, mainly to the ameba buccalis did not seem warranted either by the facts brought out in the investigations, or by the already available knowledge of the disease, inasmuch as it excluded from the process a large number of bacteria which are known to produce tissue destructions of a similar type in other regions of the body. The first few months that followed saw the rapid generalization of the treatment advocated for the destruction of this protozoon. Emetin hydrochlorid was administered to patients right and left, intra-muscularly and intra-gingivally; it was applied liberally to pyorrhea pockets all in the hope that it would prove the long-looked for panacea of destructive diseases of the peridental membrane. Gradually even the most hopeful among the advocates of emetin began to experience a disappointment based on the realization that after all a great deal more than just the administrations of emetin would be necessary to bring about, if not a cure, at least an arrest of the disease.

Of all the communications that have appeared from time to time, the one by Arthur H. Sanford, M.D., and Gordon M. New, M.D., of the Mayo Clinic, Rochester, Minn., is in our estimation deserving at this time of particular consideration by the dental profession. Before giving the results of Sanford and New's work, it might be well to recall that Smith and Barrett found the ameba buccalis in all of the forty-six cases upon which they at first reported and that none were present in seven mouths that were normal; and that Bass and Johns based their conclusions on the positive findings in three hundred gross lesions. Work carried out at the Mayo Clinic since January, 1915, has consisted in the examination of three hundred and twenty-seven cases which were classified according to degrees of evident pyorrheal infection. From this series, in cases which had practically normal mouths, that is to say, a complete absence of pyorrheal symptoms, there were eight which showed the presence of ameba or a percentage of fourteen. A group classified as slight pyorrhea showed the ameba in 43 per cent. of the cases. A group marked as moderate pyorrhea showed the ameba in 62 per cent. of the cases. A group classified as advanced pyorrhea showed 71 per cent. with ameba, and a group marked severe pyorrhea showed ameba in 80 per cent. of the cases. The authors have also established a pathogenic difference between the ameba buccalis and the ameba histolytica. If these organisms are identical a large percentage of patients with the ameba in the mouth should show ameba in the intestinal tract. Seventy-three patients with intestinal ameba and diarrhea were examined for ameba in the mouth. Of these only thirty-one showed the presence of the protozoon, a percentage of about 42; and again in a series of two hundred and fifty-four patients which showed the presence of oral ameba, the intestinal ameba was absent.

Concerning the use of emetin, the authors report that in a series of thirty-three patients with pyorrhea of varying degrees of intensity, and in which the ameba was first demonstrated, emetin was administered either hypodermically in one-third or two-third grain doses, or in tablet form two or three times daily. The result of the observations made by them is best reported literally:

''In every instance the smears failed to show the presence of the entameba within four to seven days. On the other hand, we did not observe the marked improvement locally that has been reported from many sources. Some of the patients felt much better generally, but we question if the therapeutic action of emetin does not owe its value to some general effect which has not as yet been demonstrated experimentally by the pharmacologist or recognized by the clinician. The patients were then sent to have thorough dental surgical treatment without further medical treatment, and after this an improvement was noticed. Very frequently when no ameba were found after dental treatment, by waiting four or five days the parasites could again be demonstrated. These would disappear after a few days of treatment, but only temporarily. The rather unsatisfactory results of this method of treatment suggested that we try the emetin on a group of patients that already had had repeated and thorough dental treatment by competent dentists, both locally and throughout the state. Twenty of these patients were examined by local dentists and thought suitable to test the value of emetin, since frequent and thorough instrumentation had failed to cure their pyorrhea. However, only eight of these could be induced to carry out the prescribed course of treatment. Entameba buccalis was demonstrated in all of them. They were given thorough emetin treatment and the condition of their mouths did not improve. One patient, still under observation, has had twenty-six injections of 2-3 gr. doses of emetin in addition to thorough dental attention, and there is no improvement. If emetin is the cure for pyorrhea we believe the specific action should be seen in this manner of treatment.''

Based on statistical and experimental study, the authors state that their opinion is expressed in the following conclusions:

1. Entameba buccalis is found in at least fourteen per cent. of mouths free from gingival irritation, and in relatively increasing numbers, in accordance with the degree of pyorrhea as we have classified them.

2. Clinically there is no parallelism between the presence of entameba buccalis, the parasite ameba of the mouth, and entameba histolytica, the cause of amebic dysentery.

3. We believe that before the alkaloids of ipecac can be accepted as the cure of pyorrhea alveolaris it must be established that they actually destroy the ameba in the mouth, thus removing the cause of the disease.

4. Our experiments, few as they are in number, with Sellard and Baetjer's technique of intracaecal injection convince us that entameba buccalis and entameba histolytica are not the same organism. We also hold that before entameba buccalis is called the cause of pyorrhea alveolaris its pathogenicity must be demonstrated by animal experimentation. —*Pacific Dental Gazette,* April, 1916.

